# Regional outbreak of community-associated methicillin-resistant *Staphylococcus aureus* ST834 in Japanese children

**DOI:** 10.1186/s12879-018-3646-z

**Published:** 2019-01-09

**Authors:** Yuki Uehara, Takashi Sasaki, Tadashi Baba, Yujie Lu, Eri Imajo, Yuka Sato, Shigeru Tanno, Munehiro Furuichi, Miki Kawada, Keiichi Hiramatsu

**Affiliations:** 1 Department of Infection Control Science, Juntendo University Graduate School of Medicine, 2-1-1, Hongo, Bunkyo-ku, Tokyo, 113-8421 Japan; 2 Department of Microbiology, Juntendo University Faculty of Medicine, 2-1-1, Hongo, Bunkyo-ku, Tokyo, 113-8421 Japan; 3 Department of General Medicine, Juntendo University Faculty of Medicine, 2-1-1, Hongo, Bunkyo-ku, Tokyo, 113-8421 Japan; 4 Infection Control Science Research Center, Juntendo University Graduate School of Medicine, 2-1-1, Hongo, Bunkyo-ku, Tokyo, 113-8421 Japan; 5 Department of Laboratory Medicine, Saitama City Hospital, 2460, Mimuro, Midori-ku, Saitama, 336-8522 Japan; 6 Division of Infectious Diseases, Department of Medical Subspecialties, National Center for Child Health and Development, 2-10-1, Okura, Setagaya-ku, Tokyo, 157-8535 Japan; 7 Department of Infectious Diseases, Saitama City Hospital, 2460, Mimuro, Midori-ku, Saitama, 336-8522 Japan

**Keywords:** Community-associated methicillin-resistant *Staphylococcus aureus*, Pediatrics, Purulent lymphadenitis, Regional outbreak, SaPISaitama2, Sequence type 834

## Abstract

**Background:**

Community-associated methicillin-resistant *Staphylococcus aureus* (CA-MRSA) infection has recently become a challenging problem worldwide and in Japan. We experienced 10 pediatric patients infected with CA-MRSA and hospitalized from 2011 to 2014 in a tertiary care hospital in Saitama, Japan, and assessed the characteristic of the strains using a whole genome sequencing (WGS)-based approach.

**Methods:**

CA-MRSA strains isolated from infected patients who required hospitalization for treatment were evaluated in this study. Antimicrobial susceptibility tests, molecular typing by PCR and pulse-field gel electrophoresis (PFGE) were performed to characterize MRSA strains. WGS was performed for detailed genetic analysis.

**Results:**

A total of 582 MRSA strains (35.2%) were identified among 1625 *S. aureus* strains collected during the study period. Ten MRSA strains (1.7%) were defined as CA-MRSA clinically, and all were isolated from pediatric patients. All strains mainly caused purulent lymphadenitis, were susceptible to fluoroquinolone and tetracycline, exhibited sequence type (ST) 834 or its single-locus variants and contained staphylococcal cassette chromosome *mec* (SCC*mec*) type IVc. Phylogenic analysis by PFGE and WGS revealed close relatedness of all strains, with the number of single nucleotide polymorphisms ranging from 35 to 119 by WGS. Out of the ten strains, nine possessed the genomic island SaPISaitama2 containing *tst, sec* and *sel* genes. SaPISaitama2 comprises a mosaic of genomic islands SaPIm4 and SaPIm1 harbored by a hospital-associated MRSA strain Mu50.

**Conclusions:**

This study describes a regional outbreak of ST834-related CA-MRSA in children with a unique pathogenicity island in Japan. Pediatric patient tropism of this clone could be enhanced by susceptibility to fluoroquinolones and tetracyclines, which cannot be prescribed to children.

**Electronic supplementary material:**

The online version of this article (10.1186/s12879-018-3646-z) contains supplementary material, which is available to authorized users.

## Background

Community-associated methicillin-resistant *Staphylococcus aureus* (CA-MRSA) infection is a major concern worldwide and has recently become a challenging problem in Japan [1, 2]. Multiple epidemics of CA-MRSA among Japanese children, school students and their family members have been reported, with the majority of cases appearing as skin and soft tissue infections, though there are no nationwide statistics about the prevalence of CA-MRSA in Japanese community [3–5]. MRSA strains isolated from pus or skin in outpatient settings in Japan have various molecular types, toxin genes and antimicrobial resistance profiles [6]. In addition, MRSA strains which have been recognized as community-associated, such as staphylococcal cassette chromosome *mec* (SCC*mec*) type IV sequence type (ST) 8-USA300 strains, are replacing MRSA strains in healthcare facilities [7].

We experienced an epidemic of CA-MRSA ST834 and its single-locus variants, a rare type of MRSA, in 10 pediatric inpatients over a 4-year period in a tertiary care hospital. The aim of this study was to characterize these MRSA isolates and relatedness with each other by using detailed molecular typing and determine the clinical presentation of infectious diseases caused by CA-MRSA ST834.

## Methods

### CA-MRSA isolates used in this study

Saitama City Hospital is a 567-bed tertiary care hospital in Saitama, Japan. CA-MRSA strains were collected when isolated from inpatients from January 2011 to December 2014. CA-MRSA was defined by previously reported criteria, namely, patients without any healthcare-associated risk factors such as hospitalization within 1 year of the MRSA culture date [8]. Identification of *S. aureus* isolated from the patients and susceptibility tests were initially performed by using Microscan WalkAway plus System (96-panel) and PC3.1 J (Siemens Co., Ltd., Munich, Germany). The isolates were cultured on trypticase soy agar at 37 °C for 24 h and stored in sterilized 10% skim milk (Becton, Dickinson and Co., Franklin Lakes, NJ, USA) at − 80 °C. Before storage, a single colony was suspended in 100 μl of TE buffer (10 mM Tris, 1 mM EDTA, pH 8.0) with 10 U of achromopeptidase (Wako Pure Chemical Industries, Ltd., Osaka, Japan) to reach 1.0 McFarland standard, and the suspension was incubated at 55 °C for 15 min. The supernatants were used as crude DNA extracts for conventional polymerase chain reaction (PCR) and whole genome sequencing (WGS).

### Antimicrobial susceptibility testing

Methicillin resistance and susceptibility to other antimicrobials were confirmed by the broth Microdilution Method With Dryplate ‘Eiken’ DP32, containing oxacillin, cefoxitin, gentamicin, minocycline, erythromycin, clindamycin, levofloxacin, vancomycin, teicoplanin and linezolid (Eiken Chemical Co., Ltd., Tokyo, Japan) and BBL™ Mueller Hinton II Broth (Cation-Adjusted) (Becton, Dickinson and Co.) based on Clinical and Laboratory Standards Institute breakpoints M100-S24 [9].

### Determination of SCC*mec* types and virulence genes by PCR

Determination of SCC*mec* types was performed as previously reported [10]. Staphylococcal exfoliative toxin genes (*eta*, *etb*), toxic shock syndrome toxin 1 gene (*tst*), staphylococcal enterotoxin genes (*sea*, *seb*, *sec*, *sed*, *see*, *seg*, *seh*, *sei*, *sej*) and Panton-Valentine leukocidin (PVL) genes (*lukF-PV*/*lukS-PV*) were detected by PCR-based methods [11–13].

### Pulsed-field gel electrophoresis

Pulsed-field gel electrophoresis (PFGE) by using *SmaI* digestion was performed as previously reported, and BioNumerics software version 7.5 (Applied Maths, Sint-Martens-Latem, Belgium) was used to analyze the correlations between band patterns [14]. Genetic relatedness was analyzed by the band identity using a Dice coefficient with a 1.0% band tolerance and unweighted pair group method with arithmetic mean.

### Whole genome sequencing and additional molecular typing

The Nextera XT DNA sample preparation kit (Illumina Inc., San Diego, CA, USA) was used for sample preparation for WGS. WGS was then performed on the Illumina MiSeq platform (Illumina Inc.), followed by de novo assembly using CLC Genomics Workbench version 9 (Qiagen N.V., Venlo, The Netherlands). The assembled contigs were submitted to the ResFinder 3.0 to search for drug-resistant genes, VirulenceFinder 1.5 to search for virulence factor genes, spaTyper 1.0 to identify *spa* types and MLST 1.8 for multilocus sequence typing (MLST), which are all housed on the Center for Genomic Epidemiology (CGE) website (http://www.genomicepidemiology.org//) [15–18]. For drug-resistant genes and toxin genes, 100% identity with and 100% length of the reference sequences were accepted as positive. Nucleotide sequences of quinolone resistance-determining regions (QRDRs) of type II topoisomerases (*parC* and *gyrA*) were converted into amino acid sequences and aligned with the amino acid sequences of *S. aureus* reference strain FDA209P to identify strains that had wild type QRDRs or non-synonymous (or missense) mutations [19]. For virulence factor genes, 100% identity with and > 60% length of the reference sequences were accepted as positive.

### Phylogenic analysis and comparison of the strains using whole genome sequences

Whole genome sequences of the strains were submitted to CSI phylogeny 1.4 on the CGE website to call single nucleotide polymorphisms (SNPs) and to infer a phylogeny of the 10 strains with other MRSA strains of which completed whole genome sequences were available, with *Staphylococcus argenteus* MSHR1132 set as the outgroup [20]. Phylogenic analysis was also performed among the 10 strains using Saitama2 as the reference strain [19]. Saitama2 was submitted as the representative strain of this study to the Rapid Annotations using Subsystem Technology server for gene annotation to investigate the pathogenicity islands [21, 22]. Whole genome alignment of the 10 strains was performed to investigate missing or acquisition of pathogenicity islands of the strains using the wgVISTA website (http://genome.lbl.gov/cgi-bin/WGVistaInput) [23, 24].

### Patient information

Patients’ age, sex and diagnoses were retrospectively extracted from their medical charts.

## Results

### Clinical presentation of infectious diseases and molecular typing of CA-MRSA ST834

*S. aureus* was isolated from 1625 inpatients including 582 patients with MRSA (35.8%) during the study period. Among these inpatients with MRSA, 10 patients (1.8%) were defined as having CA-MRSA. All 10 patients with CA-MRSA were hospitalized in the Department of Pediatrics. Strain and patient number, diagnosis, isolation year, sample type, results of molecular typing, toxin genes, antimicrobial susceptibilities and drug-resistant genes are listed in Table 1, shown in Additional file 1: Table S1. Detailed results of WGS are shown in Table 2, shown in Additional file 2: Table S2. Nine strains caused active infectious diseases, with eight cases of purulent lymphadenitis and one case of deep-seated infection with osteomyelitis, intramuscular abscess and bacteremia. The remaining patient was recognized as a nasal carrier of MRSA. Consequentially, all CA-MRSA 10 strains were isolated from pediatric inpatients and were classified as ST834 or its single-locus variants, and SCC*mec* type IVc. Nine strains carried *tst* and *sec* genes, which were also confirmed by the results of WGS. Two different *spa* types were found, *t*9624 (*n* = 7) and *t*1379 (*n* = 3). Antimicrobial susceptibility tests showed all 10 strains were resistant to oxacillin, cefoxitin and erythromycin and one strain was additionally resistant to gentamicin with *aac(6′)-aph(2″)*. In contrast, no strains were found to be resistant to minocycline, levofloxacin, vancomycin, teicoplanin and linezolid in this study. All QRDRs were wild type, supporting susceptibility to fluoroquinolones of these strains.

### Phylogenic analysis by pulsed-field gel electrophoresis and whole genome sequencing

**Fig. 1:**
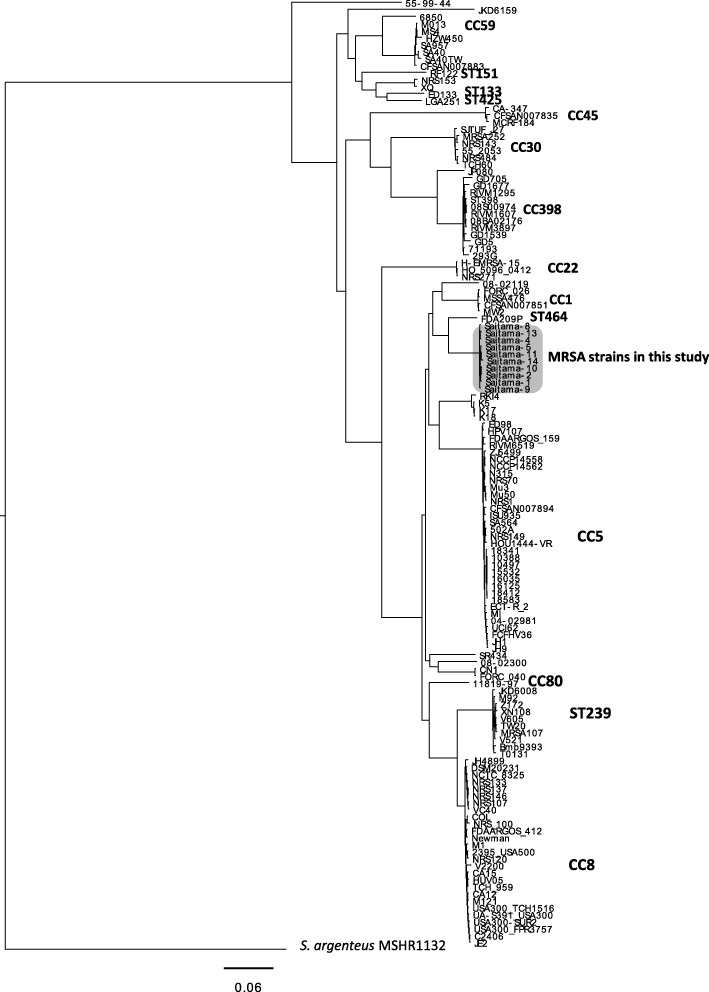
Phylogenic tree based on whole genome SNPs of the MRSA strains. *S. argenteus* MSHR1132 was used as the outgroup. The MRSA strains used in this study formed an independent cluster (gray-shaded). CC, clonal complex; MRSA, methicillin-resistant *Staphylococcus aureus*; SNPs, single nucleotide polymorphisms; ST, sequence type

**Fig. 2:**
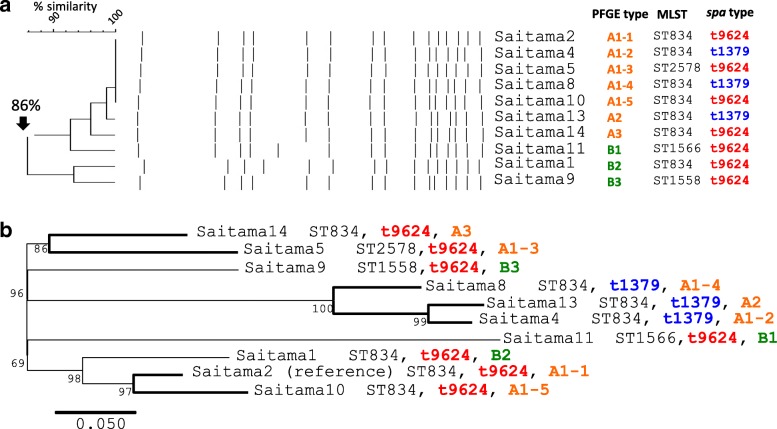
Phylogenic trees of the MRSA strains by pulsed-field gel electrophoresis and SNPs analysis. (**a**) PFGE and similarity analysis of the 10 MRSA strains. Saitama2 was used as the reference strain. Band patterns of Saitama4, − 5, − 8, − 10, − 13 and − 14 were visibly indistinguishable from that of Saitama2, and the dendrogram showed high identity with Saitama2 (PFGE type A1, 2 and 3). Saitama11, − 1 and − 9 showed different band patterns from Saitama2, however, the band differences were within three bands (PFGE type B1, 2 and 3). Saitama9 demonstrated maximum similarity to Saitama2 (86% identity). These results confirmed all 10 strains had genetic relatedness with each other. MRSA, methicillin-resistant *Staphylococcus aureus*; PFGE, pulsed-field gel electrophoresis; SNPs, single nucleotide polymorphisms. (**b**) Phylogenic tree of the 10 MRSA strains inferred with SNPs analysis. Saitama2 was used as the reference strain, and the number of SNPs ranged from 35 to 119. MRSA, methicillin-resistant *Staphylococcus aureus*; PFGE, pulsed-field gel electrophoresis; SNPs, single nucleotide polymorphisms

Figure 1 shows the result of phylogenic analysis of the MRSA strains with known complete whole genome sequences and the 10 MRSA strains in this study. Strain FDA209P was the nearest to the MRSA strains in this study but is ST464, suggesting that the MRSA strains in this study did not have any genetic relatedness with other known MRSA strains. The results of PFGE and phylogenic analysis using whole genome sequences are shown in Fig. 2. In Fig. 2a, six isolates, Saitama4, − 5, − 8, − 10, − 13 and − 14, showed PFGE band patterns that were indistinguishable from that of Saitama2. The remaining three strains, Saitama1, − 9 and − 11, also showed 86% identity with Saitama2, which suggested genetic relatedness with each other. Figure 2b shows the phylogenic tree of the 10 strains, using Saitama2 as the reference, inferred with SNP analysis using the maximum likelihood method. The three strains with *spa* type t1379 seemed to form a small cluster within all 10 strains. Compared with Saitama2, the number of SNPs of the strains ranged from 35 to 119, as shown in Table 2.

### A novel staphylococcal genomic island SaPISaitama2

**Fig. 3:**
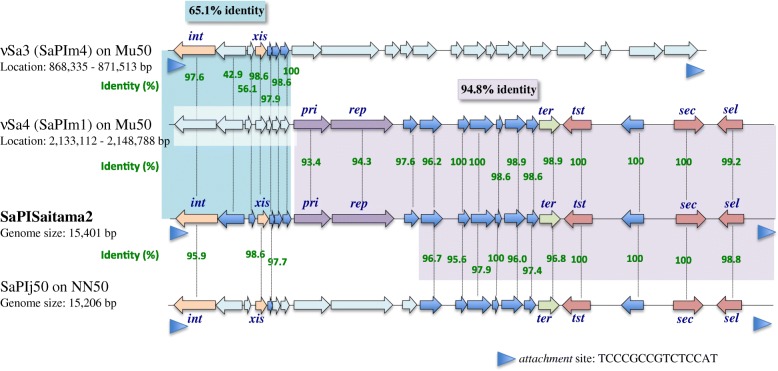
Structure of the genomic island SaPISaitama2 and comparison with genomic islands SaPIm4, SaPIm1 and SaPIj50. SaPISaitama2 was constructed with regions from SaPIm4 (ʋSa3) and SaPIm1 (ʋSa4) and could be grouped as ʋSa3, as with the integrase (*int*) of SaPIm4. SaPIj50 is also a mosaic of SaPIm4 and SaPIm1 but seems to have different motifs between the parts of SaPIm4 and SaPIm1. Coding sequences with > 80% identify with SaPISaitama2 are colored

WGS followed by gene annotation of Saitama2 indicated the possibility that Saitama2 contains a unique genomic island. Figure 3 shows the details of this new genomic island, named as SaPISaitama2, with comparison of the two genomic islands in vancomycin-intermediate *S. aureus* strain Mu50 [25]. SaPISaitama2 has a length of 15,401 bps, and contains direct repeat regions (TCCCGCCGTCTCCAT) at both ends and 21 coding sequences. Compared with genomic islands SaPIm4 and SaPIm1 in *S. aureus* Mu50, the *int* (coding integrase) sequence in SaPISaitama2 demonstrated 97.6% identity with that of SaPIm4, which was classified as ʋSa3. The 3366-bp length of the sequence from the first direct repeat to the start of *pri* (coding primase) in SaPISaitama2 showed 65.1% identity with the corresponding region in SaPIm4. The remaining 12,035-bp length of the sequence from the start of *pri* to the second direct repeat, which contained *pri*, *rep*, *ter*, *tst*, *sec* and *sel*, in SaPISaitama2 showed 94.8% identity with SaPIm1, which was classified as ʋSa4. From these results, the structure of SaPISaitama2 appears to be a mosaic of two different genomic islands that probably emerged from recombination of SaPIm4 and SaPIm1. SaPIj50 in Japanese strain CA-MRSA NN50 (ST8) was also reported as a mosaic of SaPIm4, PI T0131 and SaPIm1. However, the region containing *pri* and *rep*, possibly derived from PI T0131, did not have high identity with SaPISaitama2 [26]. The sequence of SaPISaitama2 was registered in the DNA Data Bank of Japan (DDBJ), under accession number LC315809, and the datasets analyzed during the current study are available in the DDBJ database.

### SaPISaitama2 is found in all the strains except for Saitama1

**Fig. 4:**
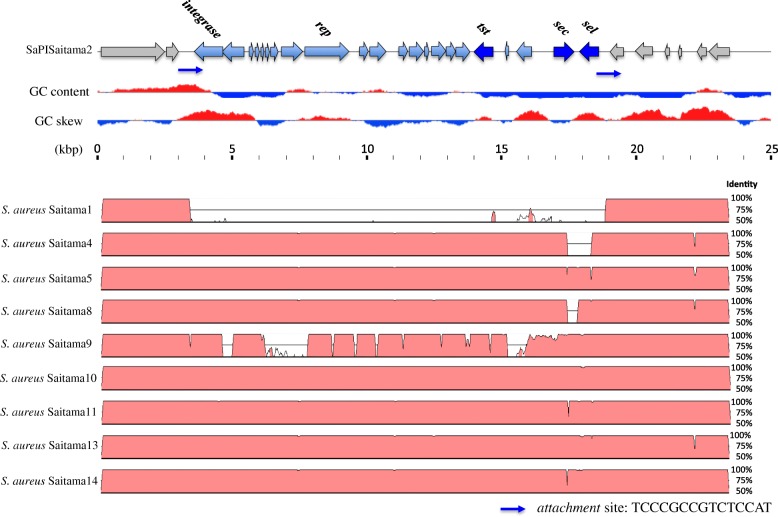
Alignment scores of pathogenicity islands on *Staphylococcus aureus* strains in this study. Saitama2 was used as the reference, and all the strains except Saitama1 seemed to contain the same pathogenicity islands of SaPISaitama2

For whole genome alignment, Saitama2 was used as the reference because Saitama2 contains complete *tst*, *sec* and *sel* genes in one assembled contig. Figure 4 shows the results of whole genome alignment of all 10 strains around the pathogenicity island. Two attachment sites were found at both ends of the pathogenicity island in all the strains except for Saitama1. SaPI of Saitama1 was deleted at the attachment sites, which was compatible with the lack of *tst* and *sec* genes in Saitama1 by PCR. In contrast, all other strains had coding sequences of *tst, sec* and *sel* between the two attachment sites*.* Although some missing regions in this genomic island were found in strains Saitama4, − 8 and − 9, as shown in Fig. 4, it is likely that they resulted from gaps in genome assembly. Based on these results, SaPISaitama2 was found to be distributed among the MRSA strains in this study except Saitama1. VirulenceFinder 1.5 could not detect *sel* of Saitama4, but *sel* was detected by alignment of the whole genome with Saitama10. Thus, *sel* of Saitama4 was described as positive in Table 2.

## Discussion

We experienced a local epidemic of CA-MRSA ST834 and its single-locus variants carrying SCC*mec* type IVc among 10 pediatric patients in Saitama city, Japan, during a 4-year period. These CA-MRSA strains mainly caused purulent lymphadenitis. In addition, they were resistant to beta-lactams and erythromycin but susceptible to fluoroquinolone and tetracycline. A novel pathogenicity region constructed by recombination of two known pathogenicity islands harbored in *S. aureus* strain Mu50, containing *tst, sec* and *sel* genes, was observed. To our knowledge, this is the first report on invasion of ST834-related CA-MRSA clone in pediatric patients in Japan.

MRSA ST834 was first registered in the MLST database from Western Australia in 2006 without any specific information of the source. We found only four articles mentioning MRSA ST834 [27–30]. Additional file 3: Table S3 summarizes the findings of these studies and our study. Reports from Kuwait and Saudi Arabia showed < 1% of MRSA strains were ST834 strains, though the strain from Saudi Arabia possessed *tst*, *sec* and *sel* genes, similar to the isolates in our study. In the report from Norway, four strains were reported as MRSA ST834 with SCC*mec* type IV and *tst*, *sec* and *sel* genes. However, the strains were isolated from individuals who had traveled to the Philippines. The authors also reported a PVL-positive strain, however, none of the strains in our study were PVL-positive. The report from Cambodia showed exceptionally high prevalence (93.4%) of MRSA ST834 by screening of children in a particular region in Cambodia in 2008 [30]. Clinical presentation of MRSA ST834 infection had not been described in these previous articles. In our study, 9 of 10 patients experienced active infectious disease and required hospitalization for treatment. The clinical presentation of infectious diseases caused by CA-MRSA ST834 was mainly purulent lymphadenitis and soft tissue infection in children, but deep-seated infection and bacteremia were also observed in one patient.

It is notable that both of our study and the report from Cambodia revealed children-tropism and regional accumulation of ST834-related MRSA [30]. Mean rates of levofloxacin and minocycline resistance of MRSA isolates from 2011 to 2014 in Japan were 87.4 and 41.0%, respectively, according to the Japan Nosocomial Infections Surveillance system organized by the Ministry of Health, Labour and Welfare of Japan [31]. Whereas in our study, all ST834-related MRSA strains showed susceptibility to tetracycline and fluoroquinolone, which are contraindicated in the field of pediatric medicine, suggesting that ST834-related MRSA could survive and increase among pediatric patients. Community- and hospital-associated MRSA reservoirs have recently begun to merge, potentially resulting from the decreasing influence of HA-MRSA in hospital environments in response to improvement of infection control measures and antimicrobial usage [32, 33]. In contrast, significant differences remain in MRSA trends in pediatric and adult patient populations, though no ST834 strains or its single-locus variants were found in Japanese children in the 2000s [32, 34–37]. In our study, ST834-related MRSA has already disseminated in children, possibly as the result of pediatric patient tropism of this clone and difference of antimicrobial use between adults and children. Further investigation of subsequent carriers in the community, both children and adults is necessary.

Conventional PFGE revealed strong genetic relatedness of the 10 MRSA strains in this study, which raised the question whether one particular clone was directly distributed in the community. Recently, phylogenic analysis using WGS data has become a popular method to analyze genetic relatedness of different strains. For instance, in outbreaks in neonatal intensive care units, direct horizontal spread of the same clone is characterized by the result of approximately 50 SNPs [38, 39], whereas regional investigation of strain USA300 reported the number of SNPs was up to 200 [40, 41]. Based on these results, the MRSA ST834 strains in this study could be recognized as causing a regional outbreak, with the number of SNPs ranging from 35 to 119. Clustering results of PFGE analysis were not completely concordant with the results of whole genome SNP clustering. WGS analysis with epidemiological information may likely replace PFGE as the gold standard for outbreak analysis in the near future.

ST834 is a double-locus variant of ST9, which has been reported as an Asian livestock-associated clone, especially swine-associated MRSA [42–44]. Previously, patients infected with MRSA ST9 linked to livestock were reported from Thailand and Taiwan [45, 46]. These MRSA ST9 strains possessed SCC*mec* XI or XII, were fluoroquinolone resistant, and had different pathogenicity genes from MRSA ST834 in this study. All reports of MRSA ST834 were from Asian countries, as described in Table 3, so further investigation of the relatedness of MRSA ST834 with livestock-associated MRSA ST9 is necessary.

Pathogenicity islands of *S. aureus* are differentiated by *int* sequences and their specific integration site on the genomes [47]. The *int* gene sequence of SaPISaitama2 is homologous to that of SaPIm4. Thus, SaPISaitama2 was recognized as ʋSa3. The region of SaPISaitama2 containing toxin genes is highly homologous to that of SaPIm1 of ʋSa4. Therefore, SaPISaitama2 was likely constructed by recombination of SaPIm4 and SaPIm1. It is difficult to determine the origin of CA-MRSA ST834, but we speculate that SaPISaitama2 is a unique pathogenicity island constructed as a mosaic of two different islands that integrated into MRSA ST834 with SCC*mec* type IVc, though this genomic island can be easily deleted according to our genome analysis, suggesting that it is not essential for evolutional strategy in ST834-related MRSA. It is still unclear whether SaPISaitama2 is directly related to disease severity because Saitama1 isolated from a patient with deep-seated infection did not possess SaPISaitama2. Other toxin genes possessed by Saitama1 were identical to other strains, suggesting that this case might have unrevealed host factors to develop severe infection by this MRSA clone.

## Conclusions

In conclusion, a regional outbreak of CA-MRSA ST834 SCC*mec* type IVc with a novel genomic island SaPISaitama2 containing *tst*, *sec* and *sel* genes was observed in Japanese children in Saitama city. This clone seemed to have advantageous antimicrobial susceptibility profile to survive among Asian children and caused lymphadenitis and skin and soft tissue infection. Continuous investigation of the distribution and clinical significance of ST834 in CA-MRSA infections in Japan is necessary.

## Additional files

Additional file 1: **Table S1.** Patient clinical information and molecular epidemiological analysis of MRSA strains ^a^ Saitama5 was found by routine nasal screening culture before scheduled orthopedic surgery. ^b^ Single-locus variant of ST834 MRSA. ^c^ SCC*mec*, staphylococcal cassette chromosome *mec*. ^d^ 100% identity with the reference sequences, but length > 60%. ^e^ VirulenceFinder 5.1 could not detect *sel* in Saitama4, but *sel* was detected by whole genome alignment. ^f^ OXA, oxacillin; FOX, cefoxitin; ERY, erythromycin; GEN, gentamicin. ^g^ QRDR, quinolone resistance-determining region. (XLSX 13 kb)

Additional file 2: **Table S2.** Detailed results of whole genome sequencing ^a^ SNPs, single nucleotide polymorphisms. (XLSX 12 kb)

Additional file 3: **Table S3.** Summary of MRSA ST834 strains from previous reports and this study ^a^ Related to traveling to the Philippines. ^b^ ST, sequence type. ^c^ ST1558, ST1566 and ST2558 are single-locus variants of MRSA ST834. ^d^ SCC*mec*, staphylococcal cassette chromosome *mec*. ^e^ PVL, Panton-Valentine leukocidin genes (*lukF-PV*/*lukS-PV*). (XLSX 10 kb)

## Abbreviations

*att*: Attachment site
CA-MRSA: Community-associated methicillin-resistant *Staphylococcus aureus*
CC: Clonal complex
CGE: Center for Genomic Epidemiology
DDBJ: DNA Data Bank of Japan
ERY: Erythromycin
FOX: Cefoxitin
GEN: Gentamicin
MLST: Multilocus sequence typing
OXA: Oxacillin
PCR: Polymerase chain reaction
PFGE: Pulsed-field gel electrophoresis
PVL: Panton-Valentine leucocidin
QRDR: Quinolone resistance-determining region
SCC*mec*: Staphylococcal cassette chromosome *mec*
SNPs: Single nucleotide polymorphisms
ST: Sequence type
WGS: Whole genome sequencing
